# A Wide QRS Complex Tachycardia with Variation of Ventriculoatrial Interval: What is the Mechanism?

**DOI:** 10.19102/icrm.2025.16021

**Published:** 2025-02-15

**Authors:** Engin Algul, Idriz Merovci, Meryem Kara, Elif Hande Ozcan Cetin, Duygu Kocyigit Burunkaya, Hamza Sunman, Ahmet Korkmaz, Firat Ozcan, Serkan Cay, Ozcan Ozeke, Ozcan Ozdemir, Dursun Aras, Serkan Topaloglu

**Affiliations:** 1 Department of Cardiology, University of Health Sciences, Ankara Etlik City Hospital, Ankara, Turkey; 2 Department of Cardiology, University Clinical Center of Kosovo, Prishtina, Kosovo; 3 Department of Cardiology, University of Health Sciences, Ankara Bilkent City Hospital, Ankara, Turkey; 4 Department of Cardiology, İstanbul Medipol University, İstanbul, Turkey

**Keywords:** Atriofascicular pathway, atrioventricular nodal reentrant tachycardia, AVNRT, Mahaim, wide complex tachycardia

## Abstract

The differential diagnosis for wide complex tachycardia includes all causes of narrow complex tachycardia with bundle branch block, all causes of narrow complex tachycardia with antegrade pre-excitation, ventricular tachycardia, and antidromic and other pre-excited reciprocating tachycardias. The variation in a specific intracardiac interval that causes a subsequent change in the tachycardia cycle length or another intracardiac interval can be diagnostic in these arrhythmias.

## Case presentation

**Figure 1:**
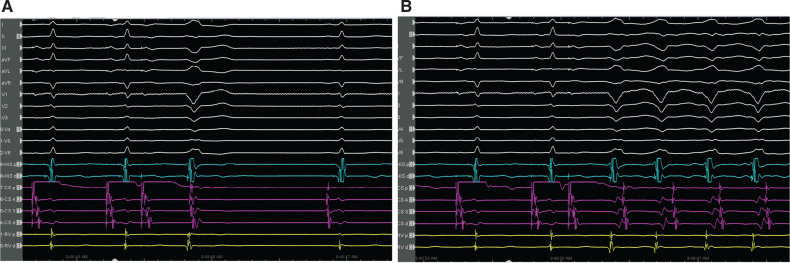
Response to programmed atrial stimulation **(A)** and the initiation of the left bundle branch block tachycardia **(B)**.

A 28-year-old woman underwent an electrophysiology study (EPS) because of episodes of palpitations with documented narrow complex tachycardia (NCT) and wide QRS complex tachycardia (WCT) on a 24-h Holter electrocardiogram (ECG). Baseline ECG showed normal sinus rhythm without ventricular pre-excitation. During the EPS, the para-Hisian pacing demonstrated the nodal response. The programmed electrical stimulation from the right atrium **(Figure 1)** showed a left bundle branch block (LBBB)-shaped WCT. There was an interesting observation in the circuit during ongoing tachycardia **(Figure 1B)**. What diagnostic information can be retrieved from the tracing?

## Discussion

Programmed atrial stimulation revealed an antegrade jump with a typical atrioventricular (AV) nodal echo beat with an LBBB-shaped QRS pattern **(Figure 1A)** and then induction of the LBBB-shaped WCT **(Figure 1B)**. The differential diagnosis for WCT includes all causes of NCT with bundle branch block, all causes of NCT with antegrade pre-excitation, ventricular tachycardia (VT), and antidromic and other pre-excited reciprocating tachycardias.^1–8^ Careful examination of the His bundle activation sequence can lead to the correct diagnosis at first glance in an otherwise highly complex diagnostic challenge.^7^ The lack of a regular H–V interval preceding each QRS complex suggests that activation does not use the orthodromic infranodal conduction system, making aberrant conduction less likely.^9^

**Figure 2:**
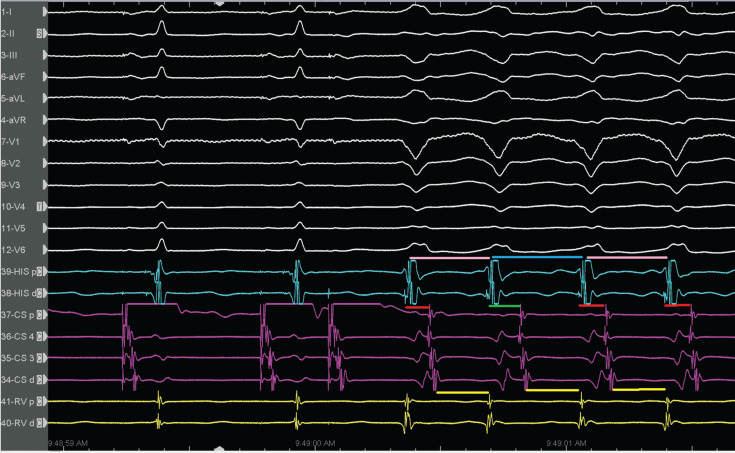
Oscillation in the cycle length and ventriculoatrial intervals. The V–A interval changes preceded and predicted the V–V intervals.

The LBBB tachycardia showed a 1:1 A–V relation and negative H–V interval, resulting in only two possibilities for the mechanism: pre-excited tachycardia (with active or passive bystander activation) or VT. The activation of the His bundle is retrograde during tachycardia, but still before ventricular activation. This is only possible in the presence of an extranodal pathway inserted in the fascicle just below the His bundle, most frequently the right bundle. An A–V interval of ≥150 ms during pre-excited tachycardia is also a fast and reliable method for detecting a decremental conducting accessory pathway.^10^ If retrograde activation of the His bundle has been determined and changes in the V–H or H–A intervals predict changes in the atrial cycle length and reset the tachycardia **(Figure 2)**, then antidromic tachycardia, either with an atrioventricular bypass tract or an atriofascicular tract, is present. Changes in the V–H or H–A intervals that cause changes in the A–A interval but do not reset the tachycardia suggest VT as the mechanism of the wide complex rhythm. Therefore, the most striking finding in the present case was that there were oscillations in the cycle length and V–A intervals **(Figure 2)**, which predicted subsequent changes in A–A intervals, implicating the retrograde conduction system in the tachycardia circuit.^4^

**Figure 3:**
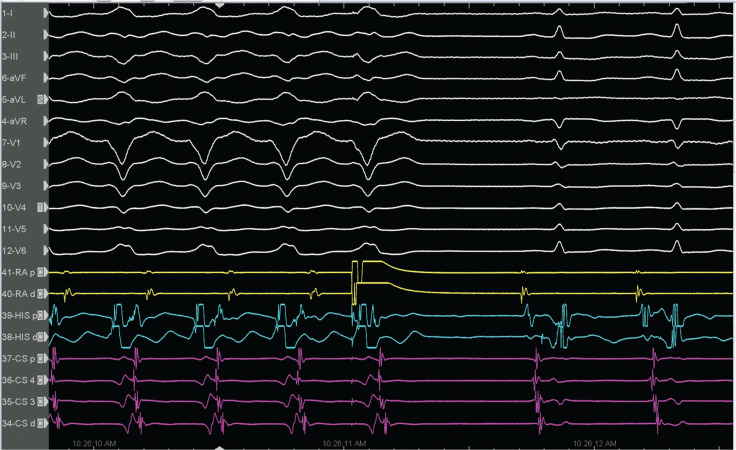
Termination response to a premature atrial beat delivered during atrioventricular nodal refractoriness.

**Figure 4:**
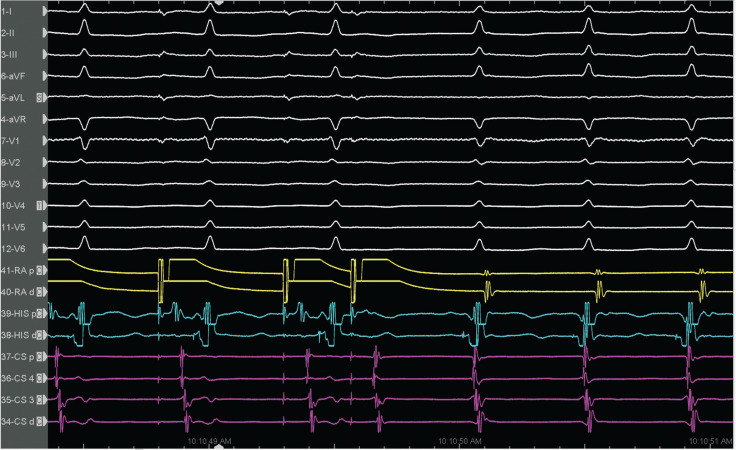
Initiation of the narrow complex tachycardia by a programmed atrial stimulation.

The rate changes in antidromic tachycardia in patients with atriofascicular fibers can be based on a shift in ventriculoatrial conduction from one bundle branch to the other by retrograde right bundle branch block.^10^ The ventriculoatrial wobble in **Figure 2** was unlikely to be due to variation in retrograde right bundle versus left bundle conduction, as the V–H interval was unchanged. There was a long–short sequence due to a fast pathway block on the first pre-excited beat, with a shortening of the anterograde conduction on the second beat. This likely causes retrograde decrement in the fast pathway (identical atrial activation sequence). Furthermore, the late-coupled premature atrial contraction resulted in tachycardia termination **(Figure 3)**, which suggests the participation of an anterogradely conducting accessory pathway, thus confirming the diagnosis of antidromic reciprocating tachycardia and ruling out pre-excited supraventricular tachycardia, nodofascicular tract, and VT.^7,11,12^ Then, a classical slow–fast atrioventricular nodal re-entrant tachycardia (AVNRT) was induced by programmed atrial stimulation **(Figure 4)**. We made the final diagnosis of dual tachycardia by typical AVNRT with atriofascicular tachycardia.^13^ After ablations at the base of Koch’s triangle for the AV nodal slow pathway and the atriofascicular accessory pathway potential at the lateral tricuspid annulus sequentially, the tachycardias were rendered non-inducible.

## References

[1] Tuncez A, Merovci I, Efe TH (2022). Transition from two wide to a narrow QRS complex tachycardia: what is the mechanism of tachycardia and transition?. J Cardiovasc Electrophysiol.

[2] Ozeke O, Cay S, Ozcan F, Topaloglu S, Aras D (2019). Electrophysiological maneuvers for concealed nodofascicular or upper common pathways: positive findings always work, but negative findings does not. Pacing Clin Electrophysiol.

[3] Ozcan Cetin EH, Kara M, Merovci I (2022). A wide QRS tachycardia with the short and long ventriculoatrial interval in the presence of an atriofascicular pathway: what is the mechanism?. J Cardiovasc Electrophysiol.

[4] Kara M, Korkmaz A, Ozeke O (2020). Wide QRS tachycardia with alternating QRS morphologies: what is the mechanism?. Pacing Clin Electrophysiol.

[5] Kara M, Korkmaz A, Karimli E (2020). A narrow QRS complex during a left bundle branch block morphology wide QRS tachycardia: a clue for manifest or bystander involvement of nodofascicular pathway?. J Cardiovasc Electrophysiol.

[6] Kara M, Cetin EHO, Korkmaz A (2022). Transient changes in QRS morphology during a narrow complex tachycardia: what is the mechanism?. J Cardiovasc Electrophysiol.

[7] Korkmaz A, Ozeke O, Cay S (2019). Response to His-refractory premature atrial complex with antegrade and retrograde septal depolarization: what is the mechanism?. J Arrhythm.

[8] Aslan AO, Merovci I, Tuncez A (2022). Widening of the QRS complex during the wide complex tachycardia: what is the mechanism?. J Cardiovasc Electrophysiol.

[9] Aras D, Ozeke O, Topaloglu S (2021). When you hear hoofbeats, look for horses, not zebras. Circulation.

[10] Sternick EB, Lokhandwala Y, Timmermans C (2009). The atrioventricular interval during pre-excited tachycardia: a simple way to distinguish between decrementally or rapidly conducting accessory pathways. Heart Rhythm.

[11] Kara M, Ozcan Cetin EH, Kocyigit Burunkaya D (2024). Wide complex tachycardia with negative precordial concordance: all that glitters is not gold. J Cardiovasc Electrophysiol.

[12] Kara M, Korkmaz A, Karimli E (2020). Unusual response to His-refractory atrial premature complex: what is the mechanism?. J Cardiovasc Electrophysiol.

[13] Marenco JP, Swygman C, Estes NA (2002). A narrow and two wide QRS complex tachycardias: what are the mechanisms?. Pacing Clin Electrophysiol.

